# Intestinal Helminths in Different Species of Rodents in North Khorasan Province, Northeast of Iran

**Published:** 2017

**Authors:** Kourosh ARZAMANI, Mitra SALEHI, Iraj MOBEDI, Amir ADINEZADE, Hamid HASANPOUR, Mohammad ALAVINIA, Jamshid DARVISH, Mohammad Reza SHIRZADI, Zeinolabedin MOHAMMADI

**Affiliations:** 1. Vector-Borne Diseases Research Center, North Khorasan University of Medical Sciences, Bojnurd, Iran; 2. Dept. of Medical Parasitology, Faculty of Medicine, Gonabad University of Medical Sciences, Gonabad, Iran; 3. Dept. of Medical Parasitology and Mycology, School of Public Health, Tehran University of Medical Sciences, Tehran, Iran; 4. Dept. of Biology, Faculty of Sciences, Ferdowsi University of Mashhad, Mashhad, Iran; 5. Rodentology Research Department (RRD), Applied Animal Institute (AAI), Ferdowsi University of Mashhad, Mashhad, Iran; 6. Zoonoses Control Department, Ministry of Health, Tehran, Iran

**Keywords:** Rodents, Helminths, Iran

## Abstract

**Background:**

Rodents are an important source of zoonotic diseases for human. The aim of this study was to determine the infectivity of rodents with intestinal helminths in North Khorasan Province, Iran.

**Methods:**

One hundred and thirteen rodents were collected using different collection methods such as kill and live traps, digging of their burrow, filling of their hiding places with water and hand net during 2011–2013. Their alimentary canals were removed in the laboratory and helminths were determined in the department of parasitology, Tehran University of Medical Sciences.

**Results:**

Thirteen species of helminths parasites were found in 13 species of rodents, including *Aspiculuris tetraptera, Hymenolepis diminuta, Nippostrongylus brasiliensis, Protospirura Seurat, Rictolaria ratti, Skrjabinitaenia lobata, Streptopharagus kuntzi, Syphacia obvelata, Taenia taeniaeformis, Trichuris muris, Cysticercus fasciolaris, Acanthocephal.* spp and *Trichuris* spp*.* Some of them were reported for the first time in new host in Iran. *S. obvelata* and *A. tetraptera* were the most frequent parasites and *P. Seurat, R. ratti* and *C. fasciolaris* were found only in one rodent.

**Conclusion:**

This is the first study to investigate the intestinal parasites in rodents in this area. Among different species identified, some of helminths were reported in new host.

## Introduction

Rodents are the most widely distributed and largest group of small mammals in the worldwide (1) and have been reported as one of the most important groups of parasite and infection reservoirs.

The helminth faunas of small mammals have been studied and documented in many countries (2–6) and the occurrence of zoonotic parasites of rodents has been studied extensively.

There are some reports on the infectivity of rodents with parasites in some areas of Iran. Three species of rodents were trapped from different localities of Khuzestan Province, southwest of Iran and *Trichosomoides crassicauda* were the most prevalent species of helminth parasites (7). Endoparasites were detected in *Meriones persicus* and *Microtus socialis* in Ardabil Province, North West of Iran (8). Endoparasites have also been isolated from *Mus musculus*, *Rattus norvegicus* and *R. rattus* in Kermanshah, west of Iran. Thus *T. muris* was the most prevalent and *S. muris* the least abundant (9). In another study, parasitic infection was observed in *Rhombomys opimus* in Golestan, adjacent Province to North Khorasan Province. The most prevalent helminth species in this study was *Dipetalonema (Acanthocheilo-nema) viteae* (10). Eleven helminths were isolated from *Apodemus sylvaticus* and *M. musculus* in Hamadan, west of Iran (11). Furthermore, 9 genera or species of the endoparasites were reported in *Tatera indica*, *Meriones hurriana*, *Gerbilus nanus* and *Meriones libycus* in Sistan and Baluchistan Province, southeast of Iran (12).

Although some investigations have been carried out on rodent parasites in Iran, there is still paucity of data regarding the parasites of rodents in northeast of Iran. This study aimed to investigate helminth infections among rodents of North Khorasan Province, northeast of Iran.

## Materials and Methods

### Study area

**Fig. 1:**
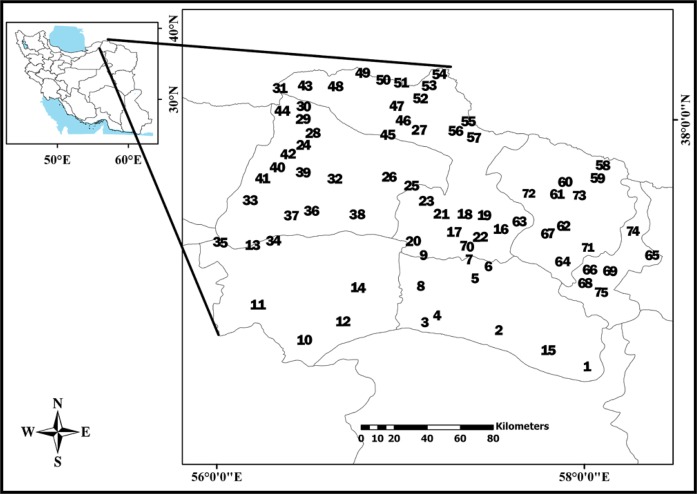
Geographical location and localities of the sampled rodents under investigation for intestinal helminths infection in North Khorasan Province, Iran, from 2011–2013

The study was conducted from 2011 to 2013 in North Khorasan Province, northeast of Iran with 36°42′ to 38°14′N and 56°03′ to 58°03′E (Fig. 1). The total area was approximately 28,179 km^2^.

### Rodent collection and Methods

The samples, which included rodent carcasses, were provided after an assessment of rodent fauna in North Khorasan Province as part of another research project carried out in Vector-borne diseases research center, Bojnurd, Iran (13). The specimens were collected using different methods including kill and live traps, digging of their burrow, filling of their hiding places with water and hand net. ArcGIS ver.9.3 software was applied for the preparation of the map of sample localities.

### Isolation of parasitic worms from rodent intestines

Rodents were anesthetized and killed by chloroform inhalation. The parasites were isolated from rodent intestines. At necropsy, their alimentary canals were removed and the contents of each part were washed in the laboratory, and the lining membrane of intestines was gently scraped with a scalpels blade and the contents were examined under a stereomicroscope. The large worms were collected from the washed materials.

The parasites were transferred to School of Public Health, Tehran University of Medical Sciences for parasites identification. Cestodes were stained with carmine acid and the nematodes were cleared in lactophenol and were identified based on morphological characteristics (14). The rodents were identified in Rodentology Research Department (RRD), Ferdowsi University of Mashhad, Iran.

### Ethical statement

Specimens’ collection was performed in accordance with the procedures approved by the Ethical Committee of North Khorasan University of Medical Sciences.

## Results

One hundred and thirteen rodents belonging to thirteen species were captured. The microscopic examination of gastrointestinal tract contents revealed the presence of thirteen species of different helminths. Fifty-eight specimens were observed which carry one or more helminth species, resulting in a prevalence of 51.3% (Table 1).

**Table 1:** Frequency of necropsied and infected rodents with intestinal helminths in North Khorasan Province, Iran from 2011–2013

**Species**	**Number of necropsied**	**Number of infected**	**Infection percent**
*Mus musculus*	10	7	70.0
*Apodemus witherbyi*	7	2	28.6
*Meriones. persicus*	24	14	58.3
*Meriones. libycus*	13	7	53.8
*Nesokia indica*	18	10	55.6
*Microtus paradoxus*	3	0	0.0
*Dryomys nitedula*	4	0	0.0
*Allactaga elater*	13	8	61.5
*Calomyscus elburzensis*	4	3	75.0
*Calomyscus mistax*	3	1	33.3
*Rhombomys opimus*	11	4	36.4
*Ellobius fuscocapillus*	1	1	100.0
*Cricetulus migratorius*	2	1	50.0
Sum	113	58	51.3

The most diversified parasites were detected in *M. persicus* (Table 2). The high percentage of infection was found in *Calomyscus elburzensis* (75%), *M. musculus* (70%) and *Allactaga elater* (61.5%), respectively. In addition, *S. obvelata* were found in one *Ellobius fuscocapillus*.

**Table 2:** Frequency of infected rodents with different intestinal helminths in North Khorasan province, Iran from 2011–2013

**Species of rodents**	**Number of infected rodents**	**Species of helminths**												
		***cysticercus fasciolaris***	***Rictolaria ratti***	***Protospirura Seurat***	***Nippostrongylus brasiliensis***	***Taenia taeniaeformis***	***Trichuris spp.***	***Streptopharagus kuntzi***	***Hymenolepis diminuta***	***Trichuris muris***	***Acanthocephal. spp.***	***skrjabinitaenia lobata***	***Aspiculuris tetraptera***	***Syphacia obvelata***
*Mus musculus*	7	+	+									+		
*Apodemus witherbyi*	2	+				+								
*Meriones persicus*	14	+		+	+	+	+		+	+			+	+
*Meriones libycus*	7	+	+	+	+			+	+	+	+			
*Nesokia indica*	10	+	+		+			+			+			
*Microtus paradoxus*	0													
*Dryomys nitedula*	0													
*Allactaga elater*	8	+	+				+							
*Calomyscus elburzensis*	3	+												
*Calomyscus mistax*	1	+	+											
*Rhombomys opimus*	4	+	+	+				+						
*Ellobius fuscocapillus*	1	+												
*Cricetulus migratorius*	1		+											

**Fig. 2:**
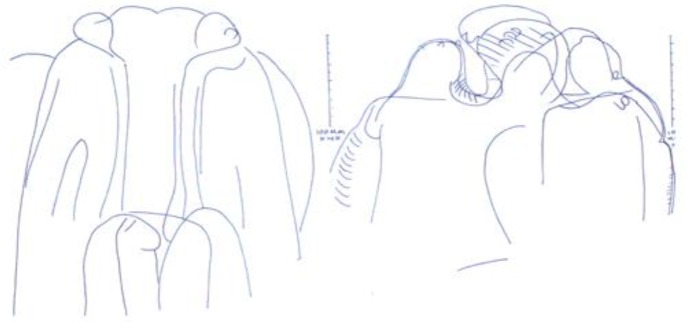
Anterior ends of *P. Seurat* with lips and papillae

**Fig. 3:**
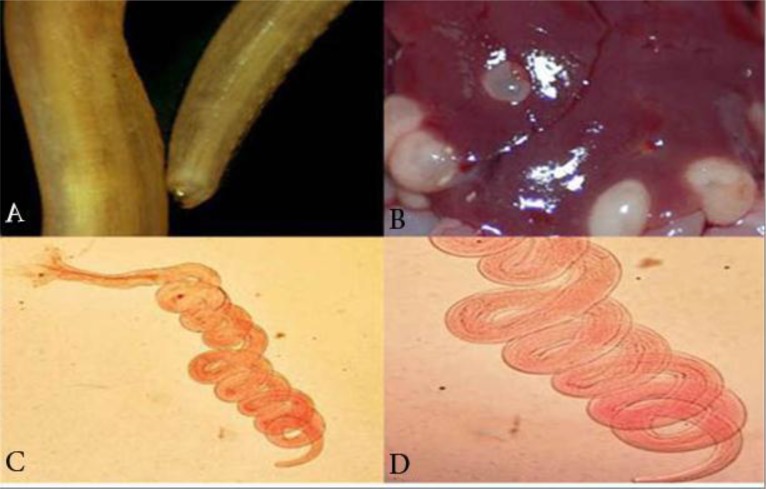
(A) *Rictulariaratti*: anterior end shows anterior combs (B) *Cysticercus fasciolaris* (C, D) Spiral shape of *Nippostrongylus brasiliensis* (Original)

*S. obvelata* and *A. tetraptera* were more frequent and *P. Seurat* (Fig. 2)*, R. ratti* (Fig. 3 (A)) and *C. fasciolaris* (Fig. 3 (B)) were found only in one specimen. The rest of the helminths parasites belong to, *H. diminuta, N. brasiliensis* (Fig. 3 (C,D))*, S. lobata, S. kuntzi, T. taeniaeformis, T. muris, Acanthocephal.* spp and *Trichuris* spp*.*

## Discussion

This study is reporting intestinal helminths from *A. elater*, *E. fuscocapillus*, *C. elburzensis* and *Calomyscus mistax* species for the first time in this part of Iran. In general, 51.3% of the rodents were infected with at least one helminth species.

The highest rates of parasitic infection were seen in *C. elburzensis* (75%)*, M. musculus* (70%) and *A. elater* (61.5%). However, parasitic infections were not found in *Microtus paradoxus* and *Dryomys nitedulaand*.

Considering the variation of parasites, eight species of helminth were found in *M. persicus. R. ratti* and *S. lobata* were seen in *M. persicus* for the first time in Iran. The result of the present study showed that this rodent species was infected with *S. obvelata*, *T. muris*, *H. diminuta*, *Trichuris* spp., *C. fasciolari*s and *Acanthocephala* spp. This result is similar with those of previous studies in Iran (8, 11).

Several researchers have isolated *Trichuris* spp*.*, *S. obvelata, S. lobata, A. tetraptera* and *Acanthocephala* spp. from *Meriones lybicus* in different parts of Iran (8, 15). In this study, *N. brasiliensis* and *S. kuntzi* were seen in *Meriones lybicus* for the first time in Iran.

*S. obvelata*, *A. tetraptera*, *Acanthocephala* spp., *S. kuntzi*, *T. taeniaeformis* and *N. brasiliensis* were reported in *Nesokia indica* for the first time in Iran. *S. obvelata* was reported in *N. indica* using histopathology method (16).

This study reported *S. kuntzi* in *Rhombomys opimus* for the first time in Iran. *S. obvelata, A. tetraptera* and *S. lobata* have been reported previously (10).

In *M. musculus* three species of helminth were found*: S. obvelata*, *A. tetraptera* and *P. Seurat*. Beside, this study is the first to report *P. Seurat* in this species in Iran. In Kermanshah and Hamadan provinces, west of Iran, *S. obvelata* was previously isolated from this species (9, 11). *S. obvelata* was isolated from *M. musculus* in Ahvaz city southwest of Iran (7). However, this rodent species had no parasitic infection in Tabriz city, northwest of Iran (17). In Brazil and Chile*, S. obvelata* and *A. tetraptera* were found in *M musculus* (18, 19). Researchers have also demonstrated *S. obvelata* in *M. musculus* in Korea (20).

Endoparasites were reported in *Cricetulus migratorius* of Tabriz city. In present study, we also isolated *A. tetraptera* from this rodent species (21).

*T. muris* and *S. obvelata* were observed in *Apodemus witherbyi* for the first time in Iran. In Khorasan Razavi Province, *Echinococcus multilocularis* infection was identified in this species (22), but in present study, this parasite could not be determined. The difference of this result is because in the first study, in addition to intestinal helminths, parasites of liver were studied using molecular method.

The species of rodents, such as *M. paradoxus, A. elater, C. elburzensis*, *E. fuscocapillus, C. mistax* and *D. nitedula*, were observed in North Khorasan Province. The following helminths were found in the above listed rodent species, with the exception of *M. paradoxus* and *D. nitedula,* for the first time in Iran: *S. obvelata* in *Ellobius fuscocapillus*; *H. diminuta, A. tetraptera*, *S. obvelata* and *T. taeniaeformis* in *A. elater, A. tetraptera* and *S. obvelata* in *C. mistax,* and *S. obvelata* in *C. elburzensis.* Besides, *Syphacia* spp. was found in *E. fuscocapillus* in Afghanistans (23). *S. obvelata* and *T. taeniaeformis* were isolated from *A. elater* in Mongolia, which is in line with the results of this study (5).

## Conclusion

Understanding of rodent parasitic fauna in different zoogeographical regions can fill the gap of information concerning the possible potentials for transmission of zoonotic helminthes to humans in the given areas. Northeastern Iran is a region with unanswered questions upon the issue. Since long time ago, the study of parasitic fauna in rodents in North East of the country has been always an attracting subject to parasitologists interested to recognize the role of rodents in zoonotic infection transmission. Further studies are required to answer some other remained questions including seasonal prevalence of each helminth, and to determine the role of paratenic and intermediate hosts involved in the lifecycle of our reported parasites. Thirteen species of parasites have been naturally found in the captured rodents in this part of Iran.
